# Screening novel genes by a comprehensive strategy to construct multiple stress-tolerant industrial *Saccharomyces cerevisiae* with prominent bioethanol production

**DOI:** 10.1186/s13068-022-02109-x

**Published:** 2022-01-21

**Authors:** Li Wang, Bo Li, Ran-Ran Su, Shi-Peng Wang, Zi-Yuan Xia, Cai-Yun Xie, Yue-Qin Tang

**Affiliations:** 1 College of Architecture and Environment, Sichuan University, No. 24 South Section 1 First Ring Road, Chengdu, 610065 Sichuan China; 2 Institute of New Energy and Low-Carbon Technology, Sichuan University, No. 24 South Section 1 First Ring Road, Chengdu, 610065 Sichuan China; 3 Engineering Research Center of Alternative Energy Materials & Devices, Ministry of Education, China, No. 24 South Section 1 First Ring Road, Chengdu, 610065 Sichuan China; 4 Sichuan Environmental Protection Key Laboratory of Organic Wastes Valorization, No. 24 South Section 1 First Ring Road, Chengdu, 610065 Sichuan China

**Keywords:** *Saccharomyces cerevisiae*, Multiple stress-tolerance, Comparative transcriptome, *ENA5*, *ASP3*, Crz1p

## Abstract

**Background:**

Strong multiple stress-tolerance is a desirable characteristic for *Saccharomyces cerevisiae* when different feedstocks are used for economical industrial ethanol production. Random mutagenesis or genome shuffling has been applied for improving multiple stress-tolerance, however, these techniques are generally time-consuming and labor cost-intensive and their molecular mechanisms are unclear. Genetic engineering, as an efficient technology, is poorly applied to construct multiple stress-tolerant industrial *S. cerevisiae* due to lack of clear genetic targets. Therefore, constructing multiple stress-tolerant industrial *S. cerevisiae* is challenging. In this study, some target genes were mined by comparative transcriptomics analysis and applied for the construction of multiple stress-tolerant industrial *S. cerevisiae* strains with prominent bioethanol production.

**Results:**

Twenty-eight shared differentially expressed genes (DEGs) were identified by comparative analysis of the transcriptomes of a multiple stress-tolerant strain E-158 and its original strain KF-7 under five stress conditions (high ethanol, high temperature, high glucose, high salt, etc.). Six of the shared DEGs which may have strong relationship with multiple stresses, including functional genes (*ASP3*, *ENA5*), genes of unknown function (*YOL162W*, *YOR012W*), and transcription factors (Crz1p, Tos8p), were selected by a comprehensive strategy from multiple aspects. Through genetic editing based on the CRISPR/Case9 technology, it was demonstrated that expression regulation of each of these six DEGs improved the multiple stress-tolerance and ethanol production of strain KF-7. In particular, the overexpression of *ENA5* significantly enhanced the multiple stress-tolerance of not only KF-7 but also E-158. The resulting engineered strain, E-158-ENA5, achieved higher accumulation of ethanol. The ethanol concentrations were 101.67% and 27.31% higher than those of the E-158 when YPD media and industrial feedstocks (straw, molasses, cassava) were fermented, respectively, under stress conditions.

**Conclusion:**

Six genes that could be used as the gene targets to improve multiple stress-tolerance and ethanol production capacities of *S. cerevisiae* were identified for the first time. Compared to the other five DEGs, *ENA5* has a more vital function in regulating the multiple stress-tolerance of *S. cerevisiae*. These findings provide novel insights into the efficient construction of multiple stress-tolerant industrial *S. cerevisiae* suitable for the fermentation of different raw materials.

**Graphical Abstract:**

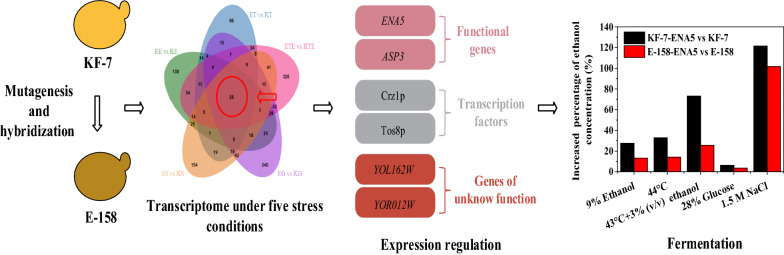

**Supplementary Information:**

The online version contains supplementary material available at 10.1186/s13068-022-02109-x.

## Highlights

Novel genes related to stress tolerance were mined by a comprehensive strategy.

Stress tolerance was improved by regulating expression of each of the novel genes.

Overexpression of *ENA5* enhanced ethanol production under all stress conditions.

Ethanol titer of the engineered strain increased by 41.35% at high temperature.

Ethanol titer of the engineered strain increased by 121.42% at salt stress condition

**Supplementary Information:**

The online version contains supplementary material available at 10.1186/s13068-022-02109-x.

## Background

Bioethanol, an eco-friendly renewable biofuel, is one of the alternatives of fossil gasoline [1]. Bioethanol production is based on the fermentation of starchy (cassava, corn, microalgae, etc.), sugary (molasses, sweet sorghum/sugarcane juice, etc.), or lignocellulosic (straw, corn cob, etc.) biomass [1, 2]. Species including *Saccharomyces cerevisiae*, *Kluyveromyces marxianus*, *Zymomonas mobilis*, and *Pichia stipites* are bioethanol producers [3–5]. *S. cerevisiae* is the main species used for industrial ethanol production due to its good fermentation performance and stress resistance [6–8]. However, *S. cerevisiae* is commonly exposed to different kinds of environmental stresses in the industrial fermentation process with different feedstocks. For example, in simultaneous saccharification and fermentation (SSF) with lignocellulosic or starchy biomass as feedstock, *S. cerevisiae* is expected to resist high temperatures (since the optimal enzymatic saccharification temperature is 45–50 °C) and high ethanol concentration [9–11]. Similarly, during very high gravity (VHG) fermentation using sugar-based raw materials (molasses, concentrated sweet sorghum juice, and sugarcane juice), strains should withstand the high concentration of sugar at the early stage and high ethanol concentration at the later stage [12]. Moreover, molasses without acid pretreatment has high salt content, which notably impedes the fermentation efficiency of *S. cerevisiae* [13, 14]. Such environmental stresses possibly cause lipid peroxidation, protein denaturation, DNA damage, cell apoptosis, etc., of *S. cerevisiae* [15, 16]. Extensive improvements of the multiple stress-tolerance and robustness of *S. cerevisiae* are of paramount to achieve high bioethanol production.

However, breeding multiple stress-tolerant industrial *S. cerevisiae* strains that perform well when using whichever feedstocks is still challenging [17]. Presently, some studies have reported that stress-tolerance of strains can be improved by random mutagenesis [18], genome shuffling [19], and genetic engineering [17]. Compared with random mutagenesis and genome shuffling, genetic engineering is of great significance because of short breeding time, clear gene targeting and clear relationship between gene and phenotype. Over the past decades, studies have reported that a number of genes were closely related to ethanol, heat, or osmosis stress tolerance of *S. cerevisiae* [9, 20–22], which can be used as the potential targets to improve stress tolerance of the strains. Based on these findings, a number of trials have been performed. For example, overexpression of *ISU1* and *JAC1* increased the ethanol tolerance [23] and overexpression of *HSF1* and *MSN2* promoted cell growth and high temperature fermentation [24]. Salt tolerance can be enhanced by over expressing *CDS1* and *CHO1* [25]. However, these studies mainly focused on the improvement of resistance to single stress (high ethanol or high temperature). To our knowledge, none of the genes linking to multiple stress-tolerance has been reported to date. Since *S. cerevisiae* strains are forced to face diverse environmental stresses during the industrial fermentation, it is vital to identify gene targets that could be engineered to improve multiple stress-tolerance of *S. cerevisiae*.

In our previous study, a *S. cerevisiae* strain E-158 was obtained by random mutagenesis and hybridization, which shows higher capability of multiple stress-tolerance than its original strain KF-7 [18]. To identify potential target genes related to multiple stress-tolerance, in the present study, the comparative transcriptome analysis was performed between strain E-158 and its original strain KF-7 under five stress conditions. Six target genes which possibly contribute to the multiple stress-tolerant phenotypes of *S. cerevisiae* were mined by a comprehensive strategy. CRISPR/Cas9 technology was used to regulate the expression of these six target genes to explore their impacts on the multiple stress-tolerance phenotypes of *S. cerevisiae*. Moreover, the stress tolerance of the engineered strains was assessed using three kinds of typical industrial feedstocks.

## Results

### Fermentation performance of original strain KF-7 and resistant strain E-158

**Fig. 1:**
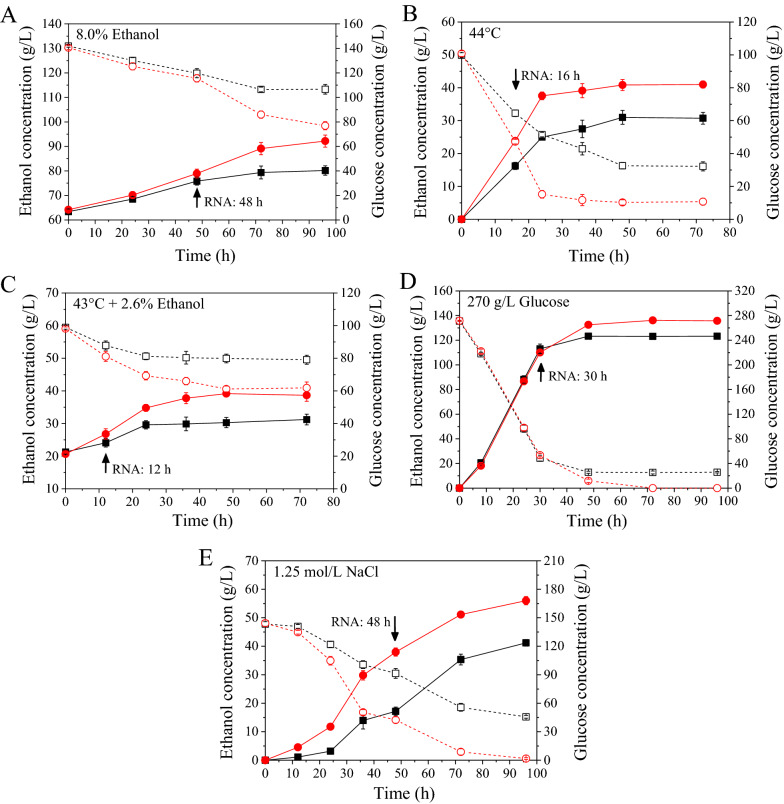
Fermentation characteristics under five stress conditions. Fermentation kinetics (**A**–**E**) of KF-7 (open squares: glucose; closed squares: ethanol) and E-158 (open circles: glucose; closed circles: ethanol) were shown. Data are averages of three independent experiments (error bars represent SD)

In our previous study, an excellent multiple stress-tolerant strain E-158 was obtained by using a strategy of random mutagenesis and hybridization [18]. The strain E-158 showed higher ethanol production and glucose consumption rates than the original strain KF-7 under five stress conditions. The final concentrations of ethanol produced by E-158 during batch fermentations were 66.89%, 33.37%, 81.02%, 10.14%, and 35.98%, respectively, higher than those of KF-7 under five stress conditions: (1) 8.0% (v/v) initial ethanol, (2) 44 °C, (3) 43 °C and 2.6% (v/v) initial ethanol, (4) 27% glucose, (5) 1.25 M NaCl (Fig. 1).

### Comprehensive strategy of mining key genes regulating stress-tolerant phenotypes of *S. cerevisiae*

**Fig. 2:**
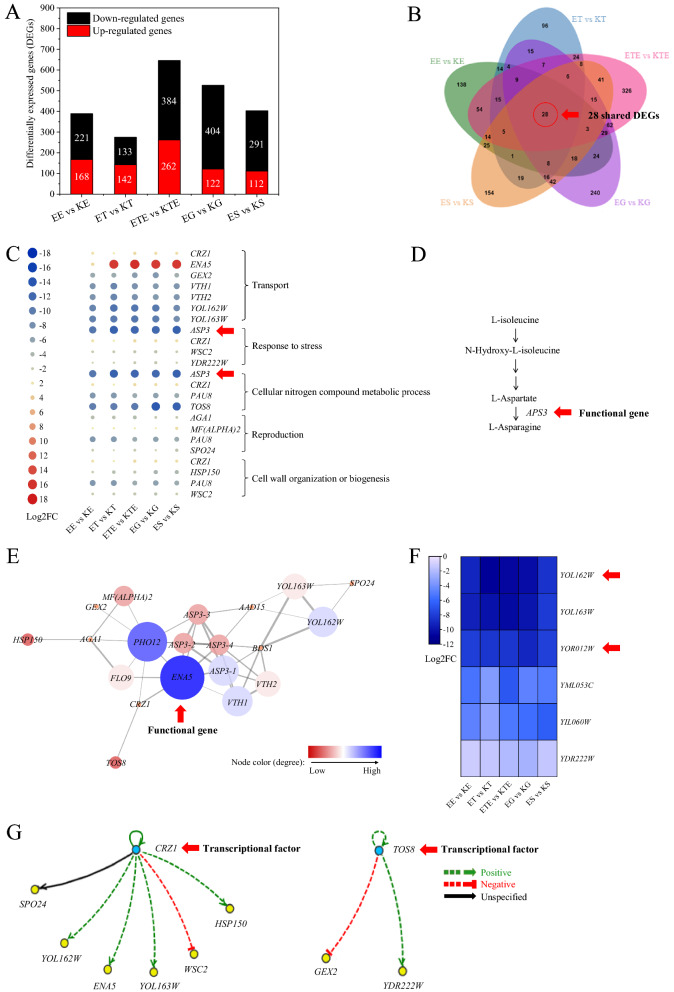
**A** comprehensive selection of key genes potentially related to the multiple stress-tolerant phenotypes. **A** DEGs under five stress conditions; **B** Venn diagram of DEGs under the five stress conditions, including 28 shared DEGs; **C** GO enrichment analyses of 28 shared DEGs; **D** the pathway of cyanoamino acid metabolism; **E** protein–protein interaction network of 28 shared DEGs. In the plot, the bluer the circle, the greater the contribution of the gene, the thicker the line, the stronger the interaction between the two genes; **F** Relative expression level of the DEGs of unknown function in 28 shared DEGs; **G** DEGs regulated by the two identified TFs in 28 shared DEGs

To mine the potential key genes governing the multiple stress-tolerant phenotypes of *S. cerevisiae*, transcriptional profiles of strains KF-7 and E-158 under five stress conditions were investigated based on RNA-seq with three biological replicates (the RNA extraction times were shown in Fig. 1, and the gene expression levels of KF-7 under same stress conditions were taken as the control group). According to differential expression analysis of RNA-seq data, hundreds of DEGs were found under each kind of stress condition (Fig. 2A).

Twenty-eight DEGs were found shared under all five stress conditions via Venn diagram (Fig. 2B, Additional file 1: Table S1), suggesting their potential contributions to multiple stress-tolerant phenotypes. GO and KEGG analyses revealed that these DEGs were involved in multiple biological processes. The functional gene *ASP3*, whose expression largely decreased (log_2_FC: − 10 ~ − 12), was involved in both processes of response to stress (GO: 0006950) and cellular nitrogen compound metabolism (GO: 0034641) (Fig. 2C). *ASP3* was also involved in cyanoamino acid metabolism (KEGG Pathway: sce00460), the only pathway significantly enriched by the KEGG analysis, suggesting *ASP3* may be one of the key genes regulating multiple-tolerant phenotypes of *S. cerevisiae* (Fig. 2D). To reveal the relationship among DEGs and to find the core regulatory target genes, protein–protein interaction network analysis was performed for the 28 shared DEGs. As shown in Fig. 2E, gene *ENA5* (log_2_FC: 2 ~ 16) was located at the core of the network and had strong relationships with other DEGs. *ENA5* encodes a P-type ATPase (extrudes Na^+^ probably in exchange for H^+^), which may assist the efflux of sodium ions, thus reducing cytotoxicity [26]. It could be one key functional gene responsible for regulating multiple-tolerant phenotypes of *S. cerevisiae*.

Six DEGs with unknown function (putative protein with unknown function in *Saccharomyces* Genome Database) in 28 shared DEGs were all significantly down-regulated (Fig. 2F). Among these six DEGs, the expression of DEGs named *YOL162W* (log_2_FC: − 8 ~ − 11), *YOL163W* (log_2_FC: − 8 ~ − 10) and *YOR012W* (log_2_FC: − 8 ~ − 10) decreased more than other three DEGs, suggesting they may have a greater impact on the tolerance phenotype [27]. Genes *YOL162W* and *YOL163W* have high sequence similarity and were proposed to have similar function [28]. Hence, *YOL162W* and *YOR012W* were selected as candidate genes to explore their effects on multiple-tolerant phenotypes of *S. cerevisiae.*

Crz1p (log_2_FC: 1 ~ 2) and Tos8p (log_2_FC: − 9 ~ − 13) were two transcriptional factors (TFs) found in the 28 shared DEGs (Fig. 2G). The regulatory relationship between these two TFs and the 28 shared DEGs was explored through YEASTRACT database. The results showed that the DEGs including *SPO24*, *ENA5*, *WSC2*, *HSP150*, *YOL162W*, and *YOL163W* among the 28 shared DEGs were potentially regulated by Crz1p. DEGs of *GEX2* and *YDR222W* were potentially regulated by Tos8p. Therefore, Crz1p and Tos8p may be key TFs regulating multiple-tolerant phenotypes of *S. cerevisiae.*

### Growth and fermentation abilities of engineered strains using original strain KF-7 as host

**Fig. 3:**
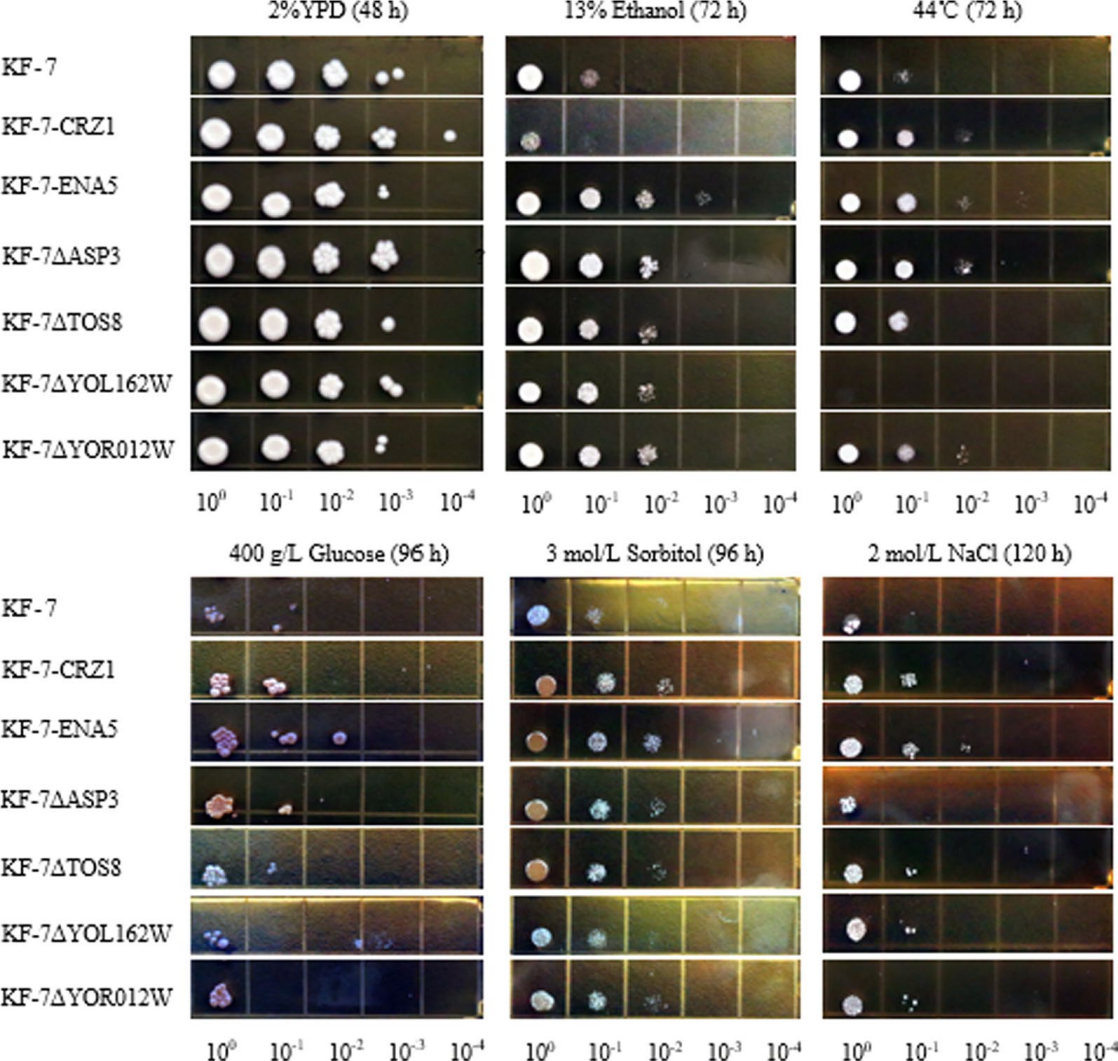
Growth abilities of engineered strains using original strain KF-7 as the host under different stress conditions

To experimentally verified whether the identified key DEGs are responsible for the multiple-tolerant phenotypes in strain E-158, using its original strain KF-7 as a host, genes *CRZ1* and *ENA5* were over expressed, and genes *ASP3*, *TOS8*, *YOL162W*, and *YOR012W* were knocked out to result in strains KF-7-CRZ1, KF-7-ENA5, KF-7ΔASP3, KF-7ΔTOS8, KF-7ΔYOL162W, and KF-7ΔYOR012W, respectively. The growth of these engineered strains under different stress conditions was compared with that of KF-7 through the spot assay (Fig. 3). Under the condition without stress, strains showed similar growth capacities. When exposed to 13% (v/v) ethanol, all strains, except strain KF-7-CRZ1, grew better than KF-7. Under the heat stress, strain KF-7ΔYOL162W did not grow when the temperature was 44 °C. However, the growth of other strains was significantly better than that of KF-7. When the spot assay was respectively performed under the osmotic stress conditions, i.e., 400 g/L glucose and 3 mol/L sorbitol, strains KF-7-CRZ1, KF-7-ENA5, KF-7ΔASP3, KF-7ΔTOS8, and KF-7ΔYOR012W grew better than KF-7, but there was no significant difference between KF-7ΔYOL162W and KF-7.

**Fig. 4:**
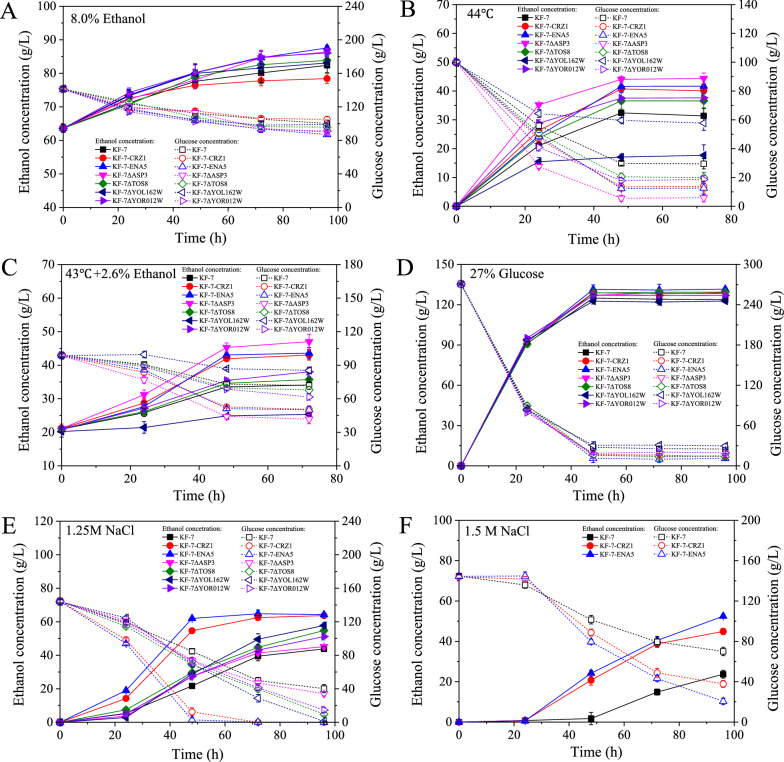
Fermentation abilities of engineered strains using original strain KF-7 as the host under five stress conditions. **A** 8.0% initial ethanol; **B** 44 °C; **C** 43 °C with 2.6% initial ethanol; **D** 270 g/L glucose; **E** 1.25 mol/L NaCl; **F** 1.5 mol/L NaCl. Data are averages of three independent experiments (error bars represent SD)

To evaluate the ability of ethanol tolerance of the engineered strains, batch fermentations were performed using YPD150 media with 8.0% (v/v) initial ethanol concentration (Fig. 4A). Except for KF-7-CRZ1 and KF-7ΔYOL162W, the other strains produced significantly (*P* < 0.05) more ethanol than KF-7 (Table 1). After 96-h fermentation, the order of the final ethanol concentrations produced by these strains was as follows: KF-7-ENA5 > KF-7ΔASP3 > KF-7ΔYOR012W > KF-7ΔTOS8 > KF-7ΔYOL162W > KF-7 > KF-7-CRZ1. The ethanol concentration of KF-7-ENA5 was the highest (23.96 ± 0.73 g/L), which was 27.57% higher than that of KF-7 (18.79 ± 2.11 g/L) (Table 1).

**Table 1:** Comparisons of fermentation performance of engineered strains using original strain KF-7 as the host

Indexes	Strains	8.0% (v/v) Initial ethanol (YPD150)	44 °C (YPD100)	43 °C + 2.6% (v/v) Initial ethanol (YPD100)	27% Glucose (YPD270)	1.25 M NaCl (YPD150)
	KF-7	18.79 ± 2.11c	31.39 ± 2.54c	13.00 ± 1.84b	123.92 ± 0.79c	43.78 ± 1.37d
Final generated ethanol concentration (g/L)	KF-7-CRZ1	14.82 ± 1.48d	40.11 ± 2.42ab	21.93 ± 1.49a	128.99 ± 1.05b	63.66 ± 1.50a
	KF-7-ENA5	23.96 ± 0.73a	41.69 ± 0.66ab	22.50 ± 1.70a	131.65 ± 0.99a	64.26 ± 1.12a
	KF-7ΔASP3	22.76 ± 1.12ab	44.37 ± 1.86a	25.91 ± 2.24a	128.19 ± 0.69b	45.16 ± 1.17d
	KF-7ΔTOS8	20.26 ± 1.57ab	36.58 ± 2.19bc	14.63 ± 1.79b	129.40 ± 0.96ab	54.78 ± 0.81bc
	KF-7ΔYOL162W	19.49 ± 0.94bc	17.70 ± 3.60d	4.24 ± 1.26c	122.80 ± 1.19c	57.77 ± 1.62b
	KF-7ΔYOR012W	22.45 ± 1.11ab	37.63 ± 1.54bc	16.94 ± 1.43b	126.95 ± 1.99bc	51.16 ± 1.33c
	KF-7	100.12 ± 4.07ab	29.51 ± 3.57b	71.80 ± 3.41b	25.09 ± 1.49a	40.18 ± 4.81a
Residual glucose (g/L)	KF-7-CRZ1	105.01 ± 3.12a	13.75 ± 3.78 cd	50.59 ± 2.80d	13.48 ± 2.58c	0.00 ± 0.00c
	KF-7-ENA5	86.88 ± 1.55d	12.62 ± 4.69 cd	50.10 ± 3.37d	10.90 ± 1.88c	0.00 ± 0.00c
	KF-7ΔASP3	89.53 ± 1.29 cd	5.89 ± 2.90d	41.76 ± 4.16d	15.05 ± 1.31bc	34.11 ± 2.72a
	KF-7ΔTOS8	95.74 ± 2.71bc	19.88 ± 3.72bc	67.85 ± 3.33bc	13.74 ± 1.91c	9.76 ± 1.88b
	KF-7ΔYOL162W	98.98 ± 1.86ab	57.75 ± 5.06a	85.36 ± 2.35a	29.66 ± 2.92a	1.36 ± 0.53c
	KF-7ΔYOR012W	90.97 ± 1.13 cd	18.56 ± 2.63c	61.66 ± 2.84c	19.48 ± 1.17b	14.66 ± 3.09b
Ethanol yield (g ethanol/g consumed glucose)	KF-7	0.45 ± 0.01a	0.45 ± 0.01bc	0.47 ± 0.01a	0.50 ± 0.01a	0.42 ± 0.02ab
	KF-7-CRZ1	0.41 ± 0.01b	0.46 ± 0.00ab	0.46 ± 0.01a	0.50 ± 0.00a	0.44 ± 0.02ab
	KF-7-ENA5	0.44 ± 0.00a	0.48 ± 0.02a	0.46 ± 0.01a	0.50 ± 0.01a	0.45 ± 0.01a
	KF-7ΔASP3	0.44 ± 0.00a	0.47 ± 0.00ab	0.46 ± 0.01a	0.50 ± 0.00a	0.41 ± 0.00bc
	KF-7ΔTOS8	0.44 ± 0.02a	0.46 ± 0.01ab	0.47 ± 0.01a	0.49 ± 0.01a	0.41 ± 0.00bc
	KF-7ΔYOL162W	0.46 ± 0.01a	0.42 ± 0.01c	0.32 ± 0.02b	0.49 ± 0.01a	0.41 ± 0.01bc
	KF-7ΔYOR012W	0.45 ± 0.01a	0.46 ± 0.01ab	0.45 ± 0.02a	0.50 ± 0.01a	0.39 ± 0.01c

The data in the table are those at the end of fermentation. Values indicate mean ± standard deviation of three biological replications. Values followed by different lowercase letters in the same column indicate significant differences at the level of *P* < 0.05 (Tukey-test) among strains. Same lowercase letters, no difference. KF-7-CRZ1: overexpression of TF Crz1p in KF-7; KF-7-ENA5: overexpression of *ENA5* in KF-7; KF-7ΔASP3: Knockout *ASP3* in KF-7; KF-7ΔTOS8: Knockout *TOS8* in KF-7; KF-7ΔYOL162W: Knockout *YOL162W* in KF-7; KF-7ΔYOR012W: Knockout *YOR012W* in KF-7

During the batch fermentations at 44 °C, the ethanol concentration of KF-7ΔYOL162W was only 56.39% of that of KF-7, but the ethanol concentrations of other strains were significantly (*P* < 0.05) higher than that of KF-7 (Fig. 4B, Table 1). Among them, KF-7ΔASP3 (44.37 ± 1.86 g/L) had the highest ethanol production, which was 41.35% higher than KF-7 (31.39 ± 2.54 g/L). Meanwhile, the ethanol concentration of KF-7ΔASP3 (25.91 ± 2.24 g/L) was 99.31% higher than that of KF-7 (13.00 ± 1.84 g/L) under two stresses of ethanol and heat (Fig. 4C, Table 1).

VHG fermentations were conducted using YP medium with 271.09 g/L glucose concentration. Except for strain KF-7ΔYOL162W, the ethanol concentrations of other strains were increased by 2.45% (KF-7ΔYOR012W) ~ 6.24% (KF-7-ENA5) compared with that of KF-7 (Fig. 4D, Table 1). All strains had improved fermentation performance when fermenting YPD150 medium supplemented with 1.25 mol/L (7.31%) NaCl (Fig. 4E). Especially, after 96-h fermentation, strains KF-7-CRZ1 and KF-7-ENA5 utilized all glucose, but the residual glucose was 43.78 ± 1.37 g/L for KF-7 (Table 1). When the concentration of NaCl was increased to 1.5 mol/L, strains KF-7-CRZ1 and KF-7-ENA5 showed high ethanol production and glucose consumption rates (Fig. 4F). The final ethanol concentrations were 52.48 ± 0.97 g/L and 44.93 ± 1.58 g/L, which were 121.42% and 89.56% higher than those of KF-7, respectively.

### Growth and fermentation abilities of engineered strains using resistant strain E-158 as host

**Fig. 5:**
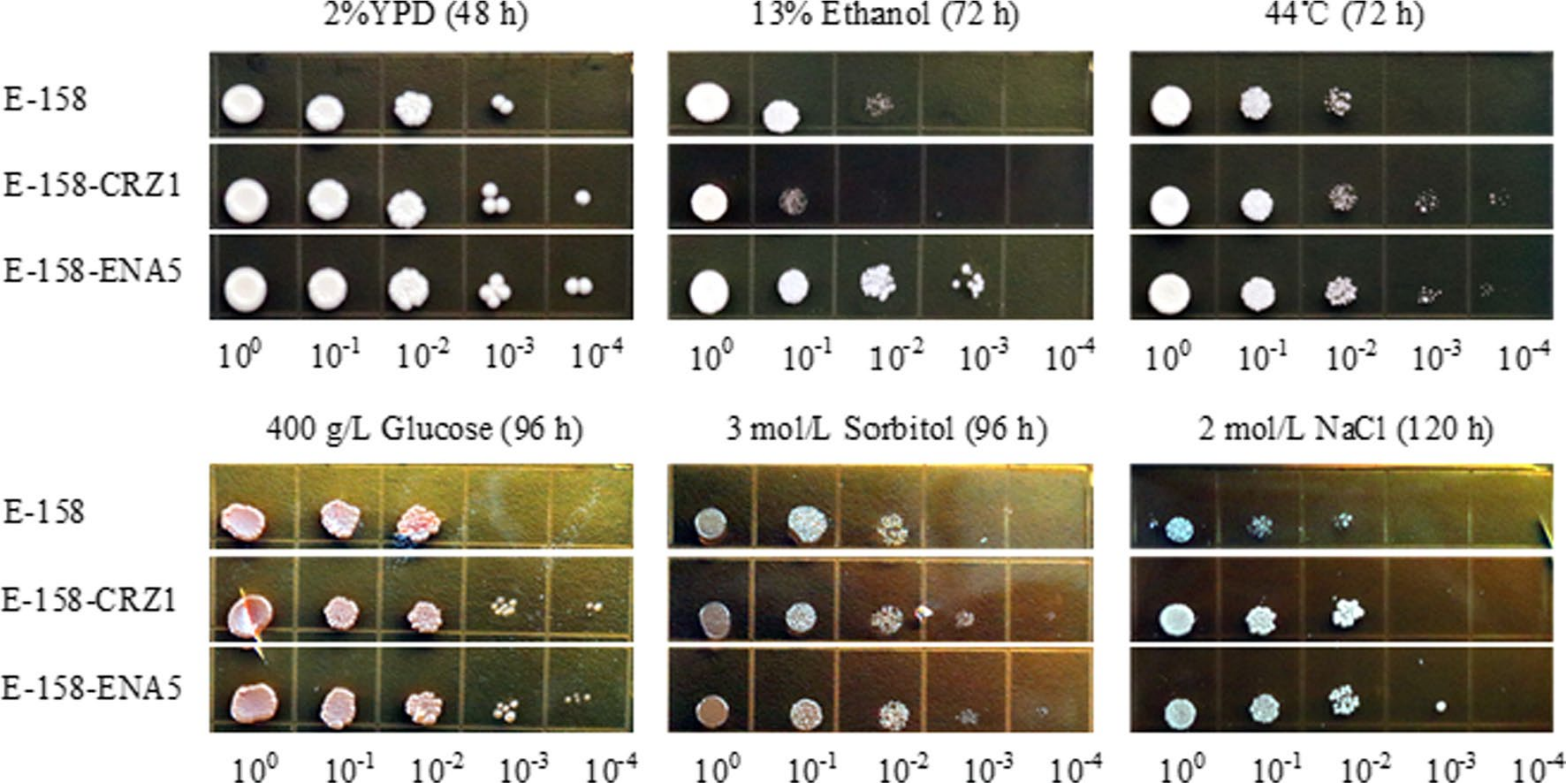
Growth abilities of engineered strains using resistant strain E-158 as the host under different stress conditions

Since the overexpression of *ENA5* or *CRZ1* significantly improved multiple stress-tolerance of the original strain KF-7, the effects of overexpression of these two genes on resistant strain E-158 were further explored. Overexpression of *ENA5* and *CRZ1* using E-158 as host resulted in strains KF-7-CRZ1 and E-158-ENA5, respectively. The growth of E-158-ENA5 was significantly better than that of E-158 in the presence of 13% (v/v) ethanol, while the growth of E-158-CRZ1 was poor (Fig. 5). E-158-CRZ1 and E-158-ENA5 grew better than E-158 under heat stress at 44 °C. Under the high osmotic conditions of 400 g/L glucose, 3 mol/L sorbitol, or 2 mol/L NaCl, the growths of E-158-CRZ1 and E-158-ENA5 were better than that of E-158. Particularly, under 2 mol/L NaCl stress, both the engineered strains showed excellent growth capacities.

**Fig. 6:**
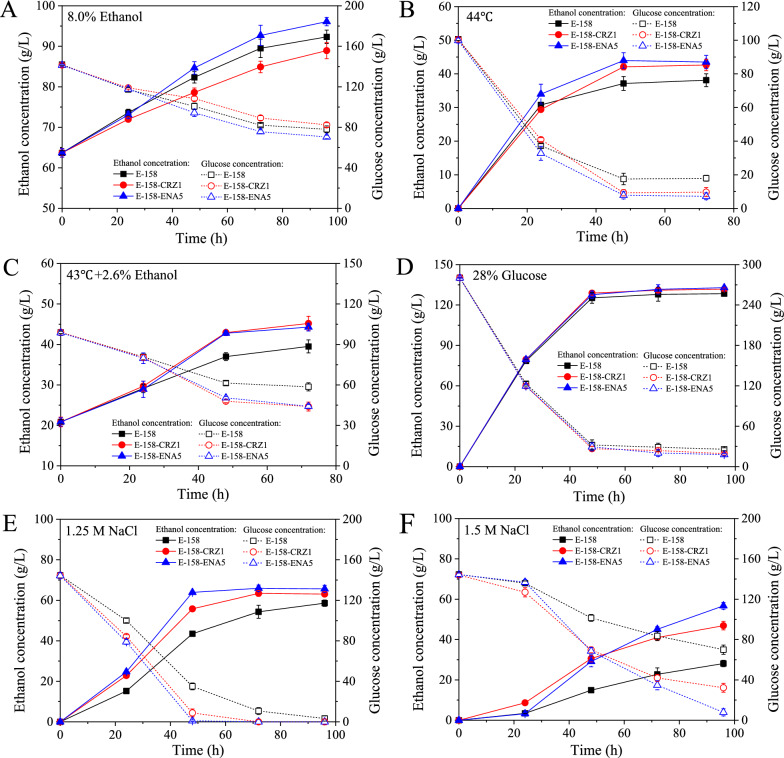
Fermentation abilities of engineered strains using resistant strain E-158 as the host under five stress conditions. **A** 8.0% initial ethanol; **B** 44 °C; **C** 43 °C with 2.6% initial ethanol; **D** 280 g/L glucose; **E** 1.25 mol/L NaCl; **F** 1.5 mol/L NaCl. Data are averages of three independent experiments (error bars represent SD)

Batch fermentations of E-158-CRZ1 and E-158-ENA5 were conducted under five stress conditions. Overexpression of *ENA5* further increased the ethanol tolerance of E-158. The ethanol produced by E-158-ENA5 (32.39 ± 1.02 g/L) was 13.09% higher than that of E-158 (28.64 ± 1.66 g/L) when YPD150 medium with 8.0% (v/v) initial ethanol concentration was fermented (Fig. 6A, Table 2). However, the ethanol concentration of strain E-158-CRZ1 was lower than that of E-158.

**Table 2:** Comparisons of fermentation performance of engineered strains using resistant strain E-158 as the host

Indexes	Strains	8.0% (v/v) Initial ethanol (YPD150)	44 °C (YPD100)	43 °C + 2.6% (v/v) Initial ethanol (YPD100)	28% Glucose (YPD280)	1.5 M NaCl (YPD150)
Final generated ethanol concentration (g/L)	E-158	28.64 ± 1.66bc	38.12 ± 1.90b	18.66 ± 1.62b	128.40 ± 1.17b	28.13 ± 1.58c
	E-158-CRZ1	25.24 ± 1.99c	42.64 ± 1.71a	24.34 ± 1.76a	131.77 ± 0.79a	46.90 ± 2.10b
	E-158-ENA5	32.39 ± 1.02a	43.52 ± 1.17a	23.43 ± 0.95a	132.90 ± 1.80a	56.73 ± 1.05a
	E-158	78.00 ± 3.40a	17.91 ± 1.72a	56.25 ± 3.00a	25.75 ± 2.22a	72.47 ± 4.53a
Residual glucose (g/L)	E-158-CRZ1	82.34 ± 2.35a	9.70 ± 2.66b	43.99 ± 3.30b	19.21 ± 1.95b	34.24 ± 4.36b
	E-158-ENA5	70.44 ± 1.74b	7.21 ± 2.03b	44.13 ± 1.40b	17.79 ± 2.79b	8.75 ± 2.54c
Ethanol yield (g ethanol/g consumed glucose)	E-158	0.45 ± 0.01ab	0.46 ± 0.00a	0.44 ± 0.00a	0.50 ± 0.00a	0.40 ± 0.01b
	E-158-CRZ1	0.43 ± 0.01b	0.47 ± 0.01a	0.44 ± 0.01a	0.50 ± 0.01a	0.43 ± 0.00a
	E-158-ENA5	0.46 ± 0.01a	0.47 ± 0.01a	0.43 ± 0.01a	0.50 ± 0.01a	0.42 ± 0.01a

The data in the table are those at the end of fermentation. Values indicate mean ± standard deviation of three biological replications. Values followed by different lowercase letters in the same column indicate significant differences at the level of *P* < 0.05 (Tukey-test) among strains. Same lowercase letters, no difference. E-158-CRZ1: Overexpression of TF Crz1p in E-158; E-158-ENA5: Overexpression of *ENA5* in E-158

Under fermentation at 44 °C, the ethanol concentrations of E-158-CRZ1 and E-158-ENA5 were increased by 11.86% and 14.17%, respectively, compared with that of E-158 (Fig. 6B, Table 2). Meanwhile, the ethanol production and glucose consumption rates of E-158-CRZ1 and E-158-ENA5 were significant higher (*P* < 0.05) than those of E-158 under the multiple stress of ethanol and heat, and the final ethanol concentrations produced by them were increased by about 30% compared with that of E-158 (Fig. 6C, Table 2).

When VHG fermentation was conducted under 280.72 g/L glucose concentration, significant (*P* < 0.05) difference in the fermentation performance among the strains was not observed (Fig. 6D). However, under the fermentation condition of 1.25 mol/L NaCl, E-158-CRZ1 and E-158-ENA5 consumed all the glucose at 72 h, which was 24 h earlier than E-158 (Fig. 6E). When the concentration of NaCl was increased to 1.5 mol/L, the final ethanol concentrations of E-158-CRZ1 and E-158-ENA5 were 46.90 ± 2.10 g/L and 56.73 ± 1.05 g/L, respectively, which were 66.73% and 101.67% higher than that of E-158 (28.13 ± 1.58 g/L) (Fig. 6F, Table 2). In conclusion, overexpression of *ENA5* improved all kinds of stress tolerance of resistant strain E-158.

### Fermentation abilities of engineered strains when fermenting pretreated straw, molasses and cassava under stress conditions

**Fig. 7:**
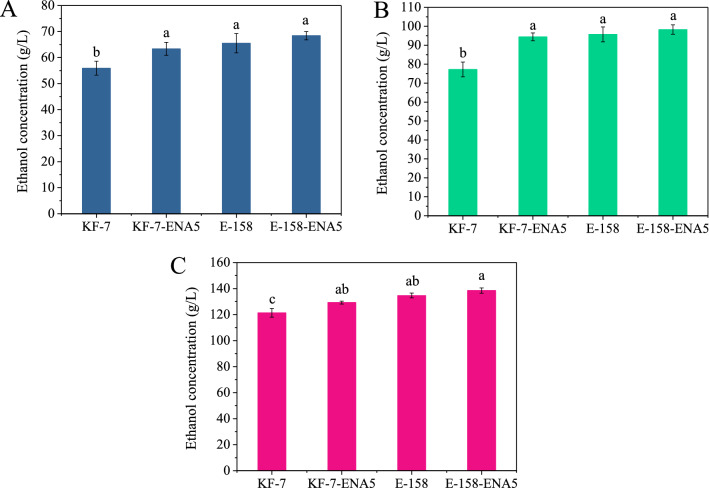
Fermentation results of engineered strains when pretreated straw, molasses, and cassava were fermented under stress conditions. **A** High-temperature SSF of pretreated straw; **B** VHG fermentation of molasses; **C** SSF of cassava with high solid content

To evaluate the potential of the strains engineered in this study for industrial applications, the fermentation abilities of strains KF-7, KF-7-ENA5, E-158, and E-158-ENA5 were assessed using pretreated straw, molasses, and cassava, which are feedstocks commonly used in industrial ethanol production (Additional file 1: Tables S2, S3, S4). By SSF of pretreated straw at 42 °C, the ethanol concentrations of KF-7-ENA5, E-158, and E-158-ENA5 were 63.35 ± 2.50 g/L, 65.50 ± 3.71 g/L, and 68.40 ± 1.59 g/L, respectively, which were increased by 13.35%, 17.19%, and 22.38% individually, compared with that of the original strain KF-7 (55.89 ± 2.68 g/L) (Fig. 7A, Additional file 1: Fig. S1A, B). The engineered strains also showed much higher ethanol yields (Additional file 1: Table S5). These results indicated that the overexpression of *ENA5* improved the fermentation capacity of the strains under high temperatures.

When the molasses with 270.91 g/L total sugar was fermented, the final ethanol concentrations of KF-7-ENA5, E-158, and E-158-ENA5 were 22.32%-27.31% higher than that of KF-7, and the strain E-158-ENA5 had the highest ethanol concentration of 98.28 g/L (Fig. 7B, Additional file 1: Fig. S1C). When cassavas with a solid content of 35% were used for SSF at 33 °C, the ethanol concentrations of strains KF-7-ENA5, E-158, and E-158-ENA5 were 6.50%, 11.01%, 14.08% respectively after 96-h fermentation, higher than that of KF-7, and the ethanol concentration produced by strain E-158-ENA5 was 138.43 g/L (Fig. 7C, Additional file 1: Fig. S1D). These results suggested that the overexpression of *ENA5* simultaneously enhanced tolerance of the strains to the heat, ethanol, and osmosis when different industrial feedstocks were fermented.

## Discussion

To find target genes that can increase the multiple stress-tolerant ability of *S. cerevisiae* suitable for various industrial feedstocks is still challenging because of the molecular regulation complexity of multiple stress-tolerant phenotypes. Presently, researchers have reported that single tolerance to ethanol or high temperature of *S. cerevisiae* can be significantly improved by over expressing or knocking out some genes or TFs [23, 24], however, researches on screening key genes responsible for enhancing the multiple stress-tolerance of industrial *S. cerevisiae* are absent. In our previous study, a multiple stress-tolerant strain E-158 was obtained by mutagenesis and hybridization using KF-7 as the starting strain [18]. In the present study, by comparing the transcriptomes of E-158 and KF-7, 28 DEGs were found shared under five stress conditions (Fig. 2). Six of them were mined and all of them were found to be associated with multiple stress-tolerant phenotypes of *S. cerevisiae*.

Among the six DEGs, *ENA5* was the most prominent in the multiple stress-tolerance improvement. To date, no report has revealed the relationship between *ENA5* and the stress tolerance phenotypes of *S. cerevisiae*. Our study revealed for the first time that the overexpression of *ENA5* could significantly improve the tolerance of *S. cerevisiae* to all kinds of stresses studied. Especially, after overexpression of *ENA5*, the ethanol concentrations of engineered strains were increased to about twofold under the conditions of 1.5 mol/L NaCl (Figs. 4F, Fig. 6F). *ENA5* is previously reported as encoding P-type ATPase that may assist the efflux of sodium ions [26, 29]. Therefore, the overexpression of *ENA5* may reduce cytotoxicity and enhance the salt tolerance of *S. cerevisiae* through assisting in the efflux of sodium ions. Genes *ENA1*、*ENA2*、*ENA5* are the members of a gene cluster encoding proteins with very similar function [30]. These genes highly expressed under high sucrose tolerance [31], which indicating ion transport is possibly important for *S. cerevisiae* to resist high osmotic stress. Nevertheless, the specific regulatory mechanism of gene *ENA5* on the multiple stress-tolerant phenotypes of *S. cerevisiae* needs to be investigated in detail in the future study.

In addition to gene *ENA5*, for the first time, gene *ASP3* was found having an important effect on high ethanol and high temperature stress tolerance phenotypes of *S. cerevisiae*. *ASP3* is a gene cluster composed of four identical genes, i.e., *ASP3-1*, *ASP3-2*, *ASP3-3*, and *ASP3-4*, which encode L-asparaginase II [32]. Asparaginase II is a periplasmic enzyme in yeast, hydrolyzing both D- and L-asparagine to aspartate and ammonium cation [32]. *ASP3* was upregulated during nitrogen starvation to facilitate the utilization of extracellular asparagine as a source of nitrogen [33]. Nitrogen starvation could promote the synthesis of lipid and/or polyhydroxybutyrate (PHB) [34, 35]. However, the molecular regulation mechanism of the deletion of *ASP3* on stress tolerance and the possible relationship between the nitrogen starvation response and the stress tolerance are still unknown, which need further investigations.

Genes of unknown function were often found expressed [36], however, no studies showed the relationship between genes of unknown function and stress-tolerant phenotypes of *S. cerevisiae*. Knocking out gene *YOR012W* of unknown function had a limited effect on the multiple stress-tolerant phenotypes of KF-7. However, knocking out *YOL162W* significantly improved the high salt tolerance while significantly reduced the high temperature resistance of the strain KF-7. Although the function of the protein encoded by *YOL162W* is unclear, *YOL162W* had a vital relationship with the high temperature and high salt tolerance phenotypes (Table 1), the underly mechanism is worthy for further investigation.

Several studies reported that TFs are important to regulate the tolerance phenotype of *S. cerevisiae* [24, 37]. However, the relationship of the two TFs, Tos8p and Crz1p, with the multiple stress tolerances was not reported to date. Tos8p was reported to be associated with chromatin and highly expressed under meiosis and cell damage [38]. In the present study, knocking out *TOS8* in original strain KF-7 increased the osmosis and high temperature tolerance phenotypes of the strain (Table 1). YEASTRACT database shows that the TF Tos8p regulates 8.0% of the genes in the genome of *S. cerevisiae*. The genes regulated by Tos8p are significantly enriched in pathways of fungal-type cell wall organization or biogenesis (GO: 0071852), cation transport (GO: 0006812), and siderophore transport (GO: 0015891). However, the molecular mechanism of the deletion of *TOS8* on the improvement of multiple stress-tolerance needs further investigations. The overexpression of *CRZ1* significantly improved the high temperature tolerance as well as the high osmosis tolerance of the strains (Figs. 4, 6). Crz1p is a zinc finger TF, which regulates 14.6% of the genes in the genome of *S. cerevisiae* based on YEASTRACT database. The genes regulated by Crz1p are significantly enriched in pathways of carbohydrate metabolic process (GO: 0005975), cell wall organization or biogenesis (GO: 0071554), and transmembrane transport (GO: 0055085). *CRZ1* has been reported able to enhance the resistance of *S. cerevisiae* to high concentrations of cations including Ca^2+^, Mn^2+^, Na^+^, and Li^+^ by regulating the genes of calcium signaling pathway [39]. Interestingly, in the present study, overexpression of *CRZ1* increased not only high salt tolerance, but also high temperature and high glucose tolerance of the strains (Tables 1, 2). Though there are reports that regulation the genes involved in carbohydrate metabolic and cell wall synthesis could effectively improve the high temperature, high ethanol, or high osmosis stress tolerance of *S. cerevisiae* [23, 24, 40], the specific molecular regulation mechanism of Crz1p on multiple stress-tolerance should be clarified in the future.

Those strains over-expressing *ENA5* showed much better ethanol production than the original strains when fermenting different raw materials. When high-temperature SSF and VHG fermentation were carried out with pretreated straw, molasses and cassava, the concentrations of ethanol produced by the engineered strains were significantly higher than those produced by the original strains and strains reported by most researchers (Fig. 7, Table 3) [13, 14, 41–47]. Considering the costs of materials, equipment, energy consumption and labor, the costs of ethanol production with straw, molasses and cassava are calculated to be about 0.60 US$, 0.62 US$, and 0.98 US$ per liter (average price of ethanol in the market is about 0.75 US$ per liter) [48–51]. Though the systematic assessments of changes of parameters including fermentation temperature, mash concentration, and ethanol concentration on the ethanol production cost when different feedstocks are used is needed. Several studies reported that if the fermentation temperature is increased by 1 °C and if the ethanol yield is increased by 10%, the cost of ethanol can be reduced by about 0.04 US$ per liter and 0.03 US$ per liter, respectively [51, 52]. This suggested that the strains engineered in the present study have good prospects for reducing ethanol production cost and hence have wide industrial applications.

**Table 3:** Comparisons of ethanol concentrations of strains when different raw materials were fermented under stress conditions

Strains	Process (Solid content)	Temperature (℃)	Substrates	Ethanol concentration (g/L)	References
Cellulose materials					
*S. cerevisiae* Angel Yeast	P-SSF (20%)	39.0	Corn stover	59.80	[41]
*S. cerevisiae* TJ14	P-SSF (/)	39.0	Microcrystalline cellulose	45.00	[42]
*K. marxianus* NRRL Y-6860	SSF (24%)	41.5	Rice straw	52.30	[43]
KF-7	P-SSF (20%)	42.0	Rice straw	55.89	This study
KF-7-ENA5	P-SSF (20%)	42.0	Rice straw	63.35	This study
E-158	P-SSF (20%)	42.0	Rice straw	65.50	This study
E-158-ENA5	P-SSF (20%)	42.0	Rice straw	68.40	This study
Molasses					
*S. cerevisiae* NCYC3233-27c	VHG (/)	35.0	Unpretreated molasses	78.90	[14]
*S. cerevisiae* UAF-1	VHG (270.0 g/L)	32.0	Acid pretreated molasses	96.00	[44]
*S. cerevisiae* SFO6	VHG (250.0 g/L)	30.0	Unpretreated molasses	55.20	[13]
KF-7	VHG (270.9 g/L)	33.0	Unpretreated molasses	77.20	This study
KF-7-ENA5	VHG (270.9 g/L)	33.0	Unpretreated molasses	94.43	This study
E-158	VHG (270.9 g/L)	33.0	Unpretreated molasses	95.71	This study
E-158-ENA5	VHG (270.9 g/L)	33.0	Unpretreated molasses	98.28	This study
Cassava					
*S. cerevisiae* CHY1011	SSF (18%)	32.0	Cassava	89.10	[45]
*S. cerevisiae* dry yeast	SSF (20%)	30.0	Cassava	71.84	[46]
*S. cerevisiae* G2-3-2	SSF (23%)	30.0	Cassava	115.77	[47]
KF-7	SSF (35%)	33.0	Cassava	121.34	This study
KF-7-ENA5	SSF (35%)	33.0	Cassava	129.23	This study
E-158	SSF (35%)	33.0	Cassava	134.70	This study
E-158-ENA5	SSF (35%)	33.0	Cassava	138.43	This study

In the previous study, we obtained strain E-158 using KF-7 as the original strain by combined techniques including ARTP mutagenesis, genome shuffling and hybridization, which took a lot of time and labor [18]. However, in the present study, the key gene *ENA5* mined by a comprehensive strategy was identified to successfully enhance multiple stresses. The engineered strain obtained by overexpression of *ENA5* in KF-7 had similar fermentation results to E-158 under all five stress conditions (Tables 1, 2), suggesting the very high effectiveness of reverse metabolic engineering employed in the present study. Directly engineering the identified key target genes significantly reduced the time and labor. Therefore, such strategy is powerful to accumulate target gene information, which can be further adopted to support the construction of excellent industrial strains.

In addition to *ENA5*, other genes investigated in the present study that contributed to stress tolerance phenotype can be applied to obtain strains with specific stress tolerance or multiple stress-tolerant strains by simultaneously regulating the expression of more than one of them. The effect of *ENA5* and other genes on the stress tolerance phenotypes can also be explored using *S. cerevisiae* strains with different genetic backgrounds, including xylose-fermenting *S. cerevisiae* strains, and even other microorganisms having industrial application potentials.

## Conclusion

In this study, six novel genes including functional genes, genes of unknown function and genes encoding TFs, were found for the first time to be related to the multiple stress-tolerant phenotypes of industrial *S. cerevisiae*. Overexpression of gene *ENA5* significantly improved the high ethanol, high temperature, high sugar, and high salt tolerance phenotypes of the original strain KF-7 and the resistant strain E-158. The fermentation performance of the engineered strains under stress conditions was much better than strains reported by most researchers. These findings provide new insights guiding the engineering of *S. cerevisiae* strains towards higher ethanol production in the presence of diverse environmental stresses.

## Materials and methods

### Strains, plasmids, primers, and media

All the strains and plasmids used and constructed in this study were listed in Table 4 [53, 54]. A flocculating industrial *S. cerevisiae* strain KF-7, which has good ethanol fermentation capacity and stress tolerance, was used as the original strain [55, 56]. The multiple stress-tolerant strain E-158 was obtained by mutagenesis and hybridization using KF-7 as the starting strain in our previous study [18]. *Escherichia coli* DH5α was used for gene cloning and manipulation. Primers and other DNA fragments used in this study were presented in Table 5 and Additional file 1: Table S6.

**Table 4:** Plasmids and strains

Plasmids and strains	Description	References
Plasmids		
Cas9-NAT	Ampr; Cas9; NAT1	[53]
pMEL13	Ampr; 2 μm origin, KanMX, gRNA-CAN1.Y	[54]
pMEL13-*CRZ1*	Ampr; 2 μm origin, KanMX, gRNA-CRZ1	This study
pMEL13-*ENA5*	Ampr; 2 μm origin, KanMX, gRNA-ENA5	This study
pMEL13-*ASP3*	Ampr; 2 μm origin, KanMX, gRNA-*ASP3*	This study
pMEL13-*TOS8*	Ampr; 2 μm origin, KanMX, gRNA-*TOS8*	This study
pMEL13-*YOL162W*	Ampr; 2 μm origin, KanMX, gRNA-*YOL162W*	This study
pMEL13-*YOR012W*	Ampr; 2 μm origin, KanMX, gRNA-*YOR012W*	This study
Strains		
KF-7	Flocculating diploid industrial *Saccharomyces cerevisiae* strain	[55]
E-158	KF-7; Random mutagenesis and hybridization	[18]
KF-7-CRZ1	KF-7; Replacement of promoter P_*CRZ1*_ to P_*TEF1*_	This study
KF-7-ENA5	KF-7; Replacement of promoter P_*ENA5*_ to P_*TEF1*_	This study
KF-7ΔASP3	KF-7; Knockout *ASP3*	This study
KF-7ΔTOS8	KF-7; Knockout *TOS8*	This study
KF-7ΔYOL162W	KF-7; Knockout *YOL162W*	This study
KF-7ΔYOR012W	KF-7; Knockout *YOR012W*	This study
E-158-CRZ1	E-158; Replacement of promoter P_*CRZ1*_ to P_*TEF1*_	This study
E-158-ENA5	E-158; Replacement of promoter P_*ENA5*_ to P_*TEF1*_	This study

**Table 5:** Target sequences used in yeast transformation

Target		Sequence
gRNA insert		
tgR-F		TGCGCATGTTTCGGCGTTCGAAACTTCTCCGCAGTGAAAGA TAAATGATC
tgR-R		GTTGATAACGGACTAGCCTTATTTTAACTTGCTATTTCTAGC TCTAAAAC
*ENA5*	CS	GATAACGTATGTACTCACTGAGG
*CRZ1*	CS	GCAGAATGTCTACTACGTCGAGG
*ASP3*	CS	GCTACGGCATGGATCAGATTAGG
*TOS8*	CS	AGAAATTACATAATAACTGTAGG
*YOL162W*	CS	AAGGTACAATGTTTAATAAATGG
*YOR012W*	CS	TTGAAACTTTTTCAGTGATTGGG
Repair fragment		
*TEF1*	F	CACACACCATAGCTTCAAAATG
	R	TTTGTAATTAAAACT
*ENA5*	RF F	CCTTCATCCTTTACATCGAGAATACGTTAACCAAATCAACCACACACCATAGCTTCAAAATG
	RF R	ATTCTTCATTATTGTTTTCTTTGACAGTTCCCTCGCTCATTTTGTAATTAAAACT
	V_P_ F	TTGTGAGGCTGATGTTTTCTTC
	V_P_ R	GCTTCTTCTGTAGTCAATGTGTGAT
*CRZ1*	RF F	GGGCTGAAAAGTACATCCGCGCATTTAACAATTGCTAAGCCACACACCATAGCTTCAAAATG
	RF R	TAGTCATGTAGGAAGCCATATTTCCGTTGCTGAATGACATTTTGTAATTAAAACT
	V_P_ F	GCTTTGACTGCACTTTAGCTTAG
	V_P_ R	TTTCCGTTGCTGAATGACAT
*ASP3*	RF F	AGAGCAAATGTTGGCTCGCTATTCTTTTGTAAGCAATCTGGTACTCACCAACCTCCAACTAGCCTGATCAGTGACTTTTCATCACACTGTGTTTTTATATAGTTCTTAGTAGTAAATATA
	RF R	TATATTTACTACTAAGAACTATATAAAAACACAGTGTGATGAAAAGTCACTGATCAGGCTAGTTGGAGGTTGGTGAGTACCAGATTGCTTACAAAAGAATAGCGAGCCAACATTTGCTCT
	V_P_ F	TATCAGACCCTTCAGCACGT
	V_P_ R	TGACACTGCTCAAGGGATAA
*TOS8*	RF F	TTTTTCAGTATAGGAAGTAATCACTGTAGAAATAAGTCAACAATAATTGCATAGAAAAAATTTTACTTTTTTCGGAATTACCTAAAATGGGTTTACGGCATAGAAGATAGATAGATTAAG
	RF R	CTTAATCTATCTATCTTCTATGCCGTAAACCCATTTTAGGTAATTCCGAAAAAAGTAAAATTTTTTCTATGCAATTATTGTTGACTTATTTCTACAGTGATTACTTCCTATACTGAAAAA
	V_P_ F	GTTCCCTTGTTTTGAAGCAC
	V_P_ R	CGAAGATTCTCACCAAAGTT
*YOL162W*	RF F	ATGTCACTTAAAATGTTATGGCAGGGGATAACAGATTACTATATATAGCCTATCTACTTGACTATGTAGAAATATGGATACAATCTCCATGTTATGTATTTTTTAAGTTTGTGAATCATT
	RF R	AATGATTCACAAACTTAAAAAATACATAACATGGAGATTGTATCCATATTTCTACATAGTCAAGTAGATAGGCTATATATAGTAATCTGTTATCCCCTGCCATAACATTTTAAGTGACAT
	V_P_ F	CTAAGCAATCACCTAAACAT
	V_P_ R	GATGTCGTACTTCTACAGCT
*YOR012W*	RF F	GAAAAAGGCAGTGACAAAAATACTAATCAGAACGTTGAAAACAAATCAATAGTTTTGATACCATCCCGAAATTAGAGGTTCAGTCAGAAAAATACTCGAAAAATATAAAACCAAAGCAGA
	RF R	TCTGCTTTGGTTTTATATTTTTCGAGTATTTTTCTGACTGAACCTCTAATTTCGGGATGGTATCAAAACTATTGATTTGTTTTCAACGTTCTGATTAGTATTTTTGTCACTGCCTTTTTC
	V_P_ F	TGCCTCATAACGTCTTGGGG
	V_P_ R	GTAGGCCGTGAATCCCTTCC

tgR-F: upstream homologous of gRNA; tgR-R: downstream homologous of gRNA; CS: complementary sequence; RF: repair fragment; Vp: verification primer; F: forward primer; R: reverse primer, “double underline” represent the PAM (NGG) site, “underline” represent homologous arm

YP medium (10 g/L yeast extract, 20 g/L peptone) containing 20 g/L glucose (YPD20) was used for cell growth. YP medium containing 150 g/L glucose (YPD150) and 8.0% (v/v) ethanol was used for ethanol-stress fermentation. YP medium containing 100 g/L glucose (YPD100) was used for heat-stress fermentation. YP medium containing 100 g/L glucose (YPD100) and 2.6% (v/v) ethanol was used for multiple-stress (ethanol and heat) fermentation. YP medium containing 270/280 g/L glucose (YPD270/280) was used for VHG-stress fermentation. YP medium containing 150 g/L glucose (YPD150) and 1.25/1.5 mol/L NaCl was used for salt-stress fermentation. YP medium containing 20 g/L glucose (YPD20), 20 g/L agar, 50 ng/mL nourseothricin (NAT), and 100 ng/mL geneticin (G418) was used for yeast transformation. LB medium (5 g/L yeast extract, 10 g/L peptone, and 10 g/L NaCl) supplemented with ampicillin (100 ng/mL) or kanamycin (100 ng/mL) was used for *E. coli* DH5α transformation.

### Batch fermentations and RNA extraction

The media and methods of batch fermentations under five stress conditions: (1) 8.0% initial ethanol; (2) 44 °C; (3) 43 °C with 2.6% initial ethanol; (4) YPD270; (5) 1.25 M NaCl were described in our previous study [18]. Strains were pre-cultivated in 100 mL YPD50 for 16 h (500 mL flasks). The cells were then collected, washed, and inoculated into fermentation media (100 mL in 300 mL flasks) with an initial cell density of OD_660_ 1.45–1.50. The flasks were incubated in thermostatic water bath, and the media was stirred (200 rpm/min) using magnetic stirrers. Broth samples were collected during fermentation and used for the analysis of the concentrations of glucose and ethanol. For each group, three replicated fermentation experiments were independently performed for the measurements.

To prepare RNA samples for transcriptomic sequencing, cells of E-158 and KF-7 were allowed to grow till logarithmic growth under five stress conditions: (1) 8.0% initial ethanol (48 h), (2) 44 °C (16 h), (3) 43 °C with 2.6% initial ethanol (12 h), (4) YPD270 (30 h), (5) 1.25 M NaCl (48 h). Then the cells were collected by centrifugation at 8000×*g* for 2 min. Total RNA was extracted from using Yeast RNA Kit (Omega Bio-Tek, USA). For each group, three replicated fermentation experiments were independently performed.

### Transcriptomic data analysis

A total of 30 mRNA samples (KF-7 and E-158 each had three parallel samples under each stress condition) were sequenced using an Illumina Novaseq 6000 platform (Shanghai Majorbio Biopharm Technology Co. Ltd. (Shanghai, China)). Library construction was conducted using the Illumina Truseq™ RNA sample prep kit. The mRNA was separated from total RNA by A-T base pairing, and then was broken into small fragments of about 300 bp by the addition of fragmentation buffer. The cDNA was synthesized using mRNA as the template. The sticky ends of the double-stranded cDNA were blunt-ended with End Repair Mix, then an A-base was added to the 3′ end for the linker to the Y-line. The resulting fragments were subjected to Illumina Novaseq sequencing. The Clean Data of each sample reached more than 6.32 Gb.

Difference in gene expression levels of one gene between KF-7 and E-158 was quantified by an index, log_2_FC, representing the logarithm to base 2 of the ratio of the RNA reads number of the gene in E158 to that in KF7. If∣log_2_FC∣ ≥ 1 and *P* < 0.05, the expression of the related gene was defined to significantly change. These analyses were performed on the online platform called Majorbio Cloud Platform (www.majorbio.com). The shared DEGs under five stress conditions were visualized by Venn-diagram (http://jvenn.toulouse.inra.fr/app/example.html). Gene Ontology (GO) enrichment of the shared DEGs was carried out with online tools developed by Princeton University (http://go.princeton.edu/cgi-bin/GOTermMapper), in which *P* ≤ 0.001 and enrichment ratio ≥ 0.1 was set as the threshold. Kyoto Encyclopedia of Genes and Genomes (KEGG) enrichment analysis of the shared DEGs was performed using the KEGG database (http://www.genome.jp/kegg/). The threshold was set to *P* ≤ 0.05 and enrichment ratio ≥ 0.1. The shared DEGs were searched in the YEASTRACT database (http://www.yeastract.com/formfindregulators.php) to find the potential TFs. The protein–protein interaction network of shared DEGs was analyzed and constructed using Cytoscape 3.7.2 software. The original sequencing data are accessed in the National Center for Biotechnology Information platform under accession number PRJNA642097.

### Strains construction

CRISPR/Case9 gene-editing technology was used to over express or knock out the genes, and the experiments were performed according to Li et al. [37]. For gRNA plasmid construction, the linearized plasmid backbone and gRNA insertion fragments were assembled to form the guideRNA (gRNA) plasmid. The linearized plasmid backbone was PCR amplified from pMEL13 plasmid using primer 6005/6006 [54]. The gRNA insert (120 bp) was composed of upstream homologous arm (tgR-F, 50 bp), downstream homologous arm (tgR-R, 50 bp), and complementary sequence (20 bp). The sequences were synthesized in GENEWIZ (Suzhou, China). The complementary sequence of gRNA was located in the promoter region for overexpression of gene *CRZ1* or *ENA5* (Additional file 1: Fig. S2A), and the complementary sequences of gRNA were located in the coding sequence (CDS) region for knocking out genes *ASP3*, *TOS8*, *YOL162W*, or *YOR012W* (Additional file 1: Fig. S2B). The gRNA sequences were designed using the E-CRISP tool at http://www.e-crisp.org/E-CRISP/ (Table 5). The gRNA was integrated into the linearized backbone using Gibson assembly according to the manufacture’s manual of Gibson Assembly® Master Mix (New England Biolabs, Beverly, MA, USA). Each plasmid was transformed into *E. coli* DH5α. After sequencing, the plasmids containing correct inserts were subsequently used for transformation.

The strength of the promoter *TEF1* (P_*TEF1*_*,* 420 bp) was high and relatively stable under five stress conditions (Additional file 1: Table S7). P_*TEF1*_ was used to replace the promoters of *CRZ1* and *ENA5*. The repair fragment was amplified using KF-7 genome as template, and it contained upstream homologous arm, downstream homologous arm, and *TEF1* sequence (Additional file 1: Fig. S2A). The repair fragments of *ASP3*, *TOS8*, *YOL162W*, and *YOR012W* were composed of upstream arm (60 bp) and downstream arm (60 bp) of the target gene CDS, which were synthesized in GENEWIZ (Suzhou, China) (Table 5, Additional file 1: Fig. S2B).

For yeast transformation, the lithium acetate method was used according to the protocol proposed by Finlayson et al. [57]. Cas9 plasmid was first transformed into KF-7 and E-158. The gRNA plasmid and repair fragment (P_*TEF1*_) were then transformed into the strains harboring Cas9 plasmid. Transformants grown on 2% YPD plate containing 0.005% NAT and 0.01% G418 were confirmed by PCR and Sanger sequencing. The removement of Cas9 and gRNA plasmids from the transformants were conducted according to Mans’ method [54]. The transformants were subjected to the following fermentation evaluation.

### Evaluation of growth and fermentation performance of engineered strains

The growth of engineered strains under different stress conditions was evaluated using YPD20 agar medium. The engineered strains were pre-cultivated in YPD50 medium for 16 h to logarithmic growth phase, and the cells were harvested by centrifugation at 8000×*g* for 2 min at 4 °C. The cells were washed twice using sterilized water and re-suspended in sterilized 0.5 mol/L ethylenediaminetetraacetic acid disodium salt (EDTA-2Na) solution with a final OD_660_ of 1.0. The solution was then serially tenfold diluted. Aliquots of 2 μL were spotted on YPD20 agar medium containing 13% (v/v) ethanol, 400 g/L glucose, 3 mol/L sorbitol, and 2 mol/L NaCl. The plates were incubated at 30 °C for 72–120 h. To examine the growth at high temperature, the plates were incubated at 44 °C for 72 h.

The fermentation capacity under different stress conditions was evaluated using both YPD medium and three different raw materials. The method of fermentation using YPD medium was the same as described in Batch fermentation and RNA extraction. The compositions of the pretreated straw, molasses, and cassava used in fermentation were shown in Additional file 1: Tables S2, S3 and S4. The fermentation using the pretreated straw was performed by pre-saccharification and SSF at 42 °C. The solid content of pretreated straw was adjusted to 20% with PBS buffer solution (pH 5). CTec3 was added at a dosage of 20 FPU/g cellulose. The slurry was pre-saccharified for 8 h at 50 °C. The pre-saccharified slurry was inoculated with pre-cultivated fresh cells (0.5 g dry weight/kg slurry), and the SSF was conducted in a thermostat water bath for 96 h at 42 °C. The VHG fermentation using molasses was conducted at 33 °C. Diluted molasses (total sugar concentration of 270.91 g/L) was inoculated with pre-cultivated fresh cells (0.5 g dry weight/L), and the VHG fermentation was conducted in a thermostat water bath for 96 h at 33 °C. High solid SSF of cassava was performed at 33 °C. Cassava slurry with 35% solid content was gelatinized at 105 °C for 15 min. The resultant gelatinized slurry was liquefied at 95 °C for 2 h by α-amylase (10 U/g starch). After cooling to room temperature, glucoamylase (160 U/g starch), pectinase (20 U/g raw materials), cellulase (10 U/g raw materials), 1 g/L KH_2_PO_4_, 0.5 g/L CaCl_2_H_2_O, 0.5 g/L MgSO_4_.7H_2_O, and 1 g/L (NH_2_)_2_CO were added. Pre-cultivated fresh cells (0.5 g dry weight/kg slurry) were inoculated. SSF was conducted in a thermostat water bath for 96 h at 33 °C. All fermentation experiments were performed three times independently.

### Analytical methods

Broth samples were diluted and filtered through 0.22 μm filters before analysis. The concentration of glucose was determined by HPLC (LC-10 ADVP, Shimadzu, Kyoto, Japan) at 25 °C, with a mobile phase of 5 mmol/L sulfuric acid at a flow rate of 0.6 mL/min. Ethanol concentration was determined using gas chromatography (GC 353B, GL Sciences, Kyoto, Japan) with an FID detector, and isopropanol was used as the internal standard. The data were expressed as the means with standard deviations. Variance analysis was conducted using Tukey-test with SPSS statistical package (SPSS Inc., Chicago, IL, USA). The level of statistical significance was set at *P* < 0.05.

## Supplementary Information

**Additional file 1.** Additional Figures and Tables.

## Data Availability

The datasets used and/or analyzed during the current study are available from the corresponding author on reasonable request. The original RNA sequencing data can be accessed through the National Center for Biotechnology Information (https://www.ncbi.nlm.nih.gov/) under project accession no. PRJNA642097.
