# Supplementary material for: Screening novel genes by a comprehensive strategy to construct multiple stress-tolerant industrial Saccharomyces cerevisiae with prominent bioethanol production
Source: Biotechnol Biofuels Bioprod. 2022 Jan 21;15:11. doi: 10.1186/s13068-022-02109-x (PMC8783499; doi:10.1186/s13068-022-02109-x)
Supplement: Supplementary file 1 — Additional file 1. Additional Figures and Tables. [file 13068_2022_2109_MOESM1_ESM.docx]

Supplementary materials

Title: Screening novel genes by a comprehensive strategy to construct multiple stress-tolerant industrial *Saccharomyces cerevisiae* with prominent bioethanol production

Author: Li Wang^1,4^, Bo Li^1,4^, Ran-Ran Su^2,3^, Shi-Peng Wang^1,4^, Zi-Yuan Xia^1,4^, Cai-Yun Xie^1,3,4,^*, Yue-Qin Tang^1,2,3,4,^*

Affiliation:

^1^College of Architecture and Environment, Sichuan University, No. 24 South Section 1 First Ring Road, Chengdu 610065, Sichuan, China

^2^Institute of New Energy and Low-carbon Technology, Sichuan University, No. 24 South Section 1 First Ring Road, Chengdu, Sichuan 610065, China

^3^Engineering Research Center of Alternative Energy Materials & Devices, Ministry of Education, China, No. 24 South Section 1 First Ring Road, Chengdu, Sichuan 610065, China

^4^Sichuan Environmental Protection Key Laboratory of Organic Wastes Valorization, No. 24 South Section 1 First Ring Road, Chengdu, Sichuan 610065, China

*Corresponding authors:

Cai-Yun Xie & Yue-Qin Tang

Telephone (Fax): +86(28)85990937, E-mail: xiecy@scu.edu.cn, tangyq@scu.edu.cn

**Fig. S1** Raw materials used in this study.

**Fig. S2** Construction of strains by CRISPR/Case9 gene-editing technology.

**Table S1** The 28 shared differentially expressed genes (DEGs) under five stress conditions.

|  |  |  | Log_2_FC |  |  |
| --- | --- | --- | --- | --- | --- |
| Genes 8.0% Ethanol 44°C 43°C+2.6% Ethanol 27% Glucose 1.25 M NaCl | | | | | |
| *MF(ALPHA)2* | 1.28 | 1.48 | 1.25 | 1.22 | 2.38 |
| *CRZ1* | 2.06 | 1.38 | 2.55 | 2.39 | 2.22 |
| *ENA5* | 2.65 | 15.42 | 16.32 | 15.28 | 15.51 |
| *AGA1* | -3.20 | -2.99 | -3.32 | -2.03 | -1.98 |
| *WSC2* | -1.86 | -2.11 | -2.29 | -2.62 | -1.86 |
| *BDS1* | -11.98 | -12.14 | -12.85 | -11.99 | -11.24 |
| *ASP3-1* | -10.44 | -12.77 | -12.25 | -12.00 | -12.80 |
| *ASP3-3* | -10.44 | -12.77 | -12.25 | -12.00 | -12.80 |
| *ASP3-2* | -10.44 | -12.77 | -12.25 | -12.00 | -12.80 |
| *ASP3-4* | -10.44 | -12.77 | -12.25 | -12.00 | -12.80 |
| *TOS8* | -9.79 | -9.87 | -10.14 | -13.73 | -12.84 |
| *YOL163W* | -9.39 | -10.11 | -10.48 | -9.90 | -8.12 |
| *YOL162W* | -9.17 | -10.95 | -10.59 | -10.33 | -8.55 |
| *YOR012W* | -7.93 | -8.33 | -8.36 | -9.07 | -7.86 |
| *AAD15* | -7.57 | -7.11 | -7.19 | -8.13 | -8.10 |
| *VTH1* | -7.47 | -9.02 | -7.69 | -7.01 | -7.31 |
| *VTH2* | -7.47 | -9.02 | -7.69 | -7.01 | -7.31 |
| *PAU8* | -7.15 | -7.00 | -5.10 | -6.21 | -5.12 |
| *YDR261W-A* | -5.79 | -1.81 | -4.95 | -4.81 | -5.48 |
| *YML053C* | -5.42 | -3.50 | -6.68 | -4.66 | -5.02 |
| *FLO9* | -5.38 | -7.17 | -4.99 | -5.87 | -8.78 |
| *GEX2* | -5.21 | -5.14 | -6.55 | -7.10 | -4.68 |
| *YIL060W* | -4.67 | -3.10 | -5.24 | -5.67 | -6.40 |
| *PHO12* | -2.91 | -2.19 | -2.64 | -2.14 | -2.91 |
| *SPO24* | -1.78 | -1.07 | -1.92 | -1.29 | -2.52 |
| *HSP150* | -1.75 | -3.07 | -2.41 | -4.08 | -3.81 |
| *MAE1* | -1.30 | -1.37 | -5.08 | -1.66 | -2.27 |
| *YDR222W* | -1.17 | -1.45 | -2.02 | -2.33 | -1.39 |

**Table S2** The components of pretreated straw.

| [Index](javascript:;)s | Results based on wet weight |
| --- | --- |
| Moisture content | 75.45±0.11% |
| pH | 7.48±0.02 |
| Cellulose | 204.78±13.79 (g/kg) |
| Hemicellulose | 11.57±0.20 (g/kg) |
| [Formic](javascript:;) [acid](javascript:;) | 0.005±0.005 (g/kg) |
| Acetic acid | 0.008±0.002 (g/kg) |
| Levulinic acid | 0.008±0.005 (g/kg) |
| Total phenols | 0.046±0.005 (g/kg) |

**Table S3** The components of molasses.

| [Index](javascript:;)s | Results based on wet weight |
| --- | --- |
| [Total](javascript:;) [sugar](javascript:;) | 457.2±6.3 (mg/g) |
| Ash content | 101±0.1 (mg/g) |
| K^+^ | 34.8±0.5 (mg/g) |
| Na^+^ | 0.8±0.2 (mg/g) |
| Ca^2+^ | 0.1±0.1 (mg/g) |
| Mg^2+^ | ND |
| Fe^2+^ | ND |
| NH_4_^+^ | 1.5±0.1 (mg/g) |
| SO_4_^2-^ | 20.9±1.0 (mg/g) |
| Cl^-^ | 23.3±0.0 (mg/g) |
| C | 35.82±0.0% |
| H | 5.58±0.1% |
| N | 2.46±0.0% |
| S | ND |

**Table S4** The components of cassava.

| [Index](javascript:;)s | Results based on dry weight (%) |
| --- | --- |
| Starch | 83.52±2.18 |
| Cellulose | 1.82±0.10 |
| Lignin | 1.47±0.13 |
| Ash content | 2.08±0.21 |
| Protein | 0.02±0.00 |

**Table S5** Fermentation results of strains when pretreated straw, molasses, and cassava were fermented under stress conditions.

| Raw materials | Strains | Total sugar content (g/L) | Ethanol concentration (g/L) | Ethanol yield (Based on the total sugar) |
| --- | --- | --- | --- | --- |
| Pretreated straw |  |  |  |  |
|  | KF-7 | 182.77±10.11 | 55.89±2.68 | 0.60±0.01 |
|  | KF-7-ENA5 | 182.77±10.11 | 63.35±2.5 | 0.68±0.01 |
|  | E-158 | 182.77±10.11 | 65.5±3.71 | 0.70±0.01 |
|  | E-158-ENA5 | 182.77±10.11 | 68.4±1.59 | 0.73±0.01 |
| Molasses |  |  |  |  |
|  | KF-7 | 270.91±5.87 | 77.20±3.92 | 0.56±0.02 |
|  | KF-7-ENA5 | 270.91±5.87 | 94.43±2.07 | 0.68±0.01 |
|  | E-158 | 270.91±5.87 | 95.71±3.91 | 0.69±0.02 |
|  | E-158-ENA5 | 270.91±5.87 | 98.28±2.47 | 0.71±0.01 |
| Cassava |  |  |  |  |
|  | KF-7 | 321.55±6.53 | 121.34±3.32 | 0.74±0.02 |
|  | KF-7-ENA5 | 321.55±6.53 | 129.23±1.08 | 0.79±0.01 |
|  | E-158 | 321.55±6.53 | 134.70±1.87 | 0.82±0.01 |
|  | E-158-ENA5 | 321.55±6.53 | 138.43±2.06 | 0.84±0.01 |

Ethanol concentration: Ethanol concentration after 96 h fermentation; Ethanol yield = Ethanol concentration / (Total sugar content 🞨 0.51)

**Table S6** Sequence of the promoter of TEF1.

| Name | Sequence（5’~3’） |
| --- | --- |
| P*_TEF1_* | CACACACCATAGCTTCAAAATGTTTCTACTCCTTTTTTACTCTTCCAGATTTTCTCGGACTCCGCGCATCGCCGTACCACTTCAAAACACCCAAGCACAGCATACTAAATTTCCCCTCTTTCTTCCTCTAGGGTGTCGTTAATTACCCGTACTAAAGGTTTGGAAAAGAAAAAAGAGACCGCCTCGTTTCTTTTTCTTCGTCGAAAAAGGCAATAAAAATTTTTATCACGTTTCTTTTTCTTGAAAATTTTTTTTTTTGATTTTTTTCTCTTTCGATGACCTCCCATTGATATTTAAGTTAATAAACGGTCTTCAATTTCTCAAGTTTCAGTTTCATTTTTCTTGTTCTATTACAACTTTTTTTACTTCTTGCTCATTAGAAAGAAAGCATAGCAATCTAATCTAAGTTTTAATTACAAA |

**Table S7** FPKM values of genes under different stress conditions.

| Genes | Strains | 8.0% Ethanol | 44℃ | 2.6% Ethanol+43℃ | 270 g/L Glucose | 1.25 M NaCl |
| --- | --- | --- | --- | --- | --- | --- |
| *TEF1* | KF-7 | 10579.70±189.25 | 3095.10±234.13 | 6638.31±393.16 | 14267.46±650.38 | 3894.25±162.30 |
|  | E-158 | 10222.68±203.56 | 2998.31±216.81 | 5704.50±429.50 | 7735.50±139.77 | 5757.41±542.63 |
| *CRZ1* | KF-7 | 24.34±4.89 | 58.97±11.19 | 28.32±3.89 | 19.34±2.89 | 26.83±1.31 |
|  | E-158 | 110.53±9.14 | 178.72±4.31 | 189.74±10.17 | 155.00±6.17 | 133.55±5.48 |
| *ENA5* | KF-7 | 18.78±5.57 | 0.00±0.00 | 0.00±0.00 | 0.00±0.00 | 0.00±0.00 |
|  | E-158 | 128.53±2.25 | 139.26±19.83 | 351.65±7.73 | 206.61±8.38 | 171.58±10.32 |
| *ASP3* | KF-7 | 29.34±1.41 | 70.26±1.31 | 52.59±5.64 | 31.10±2.31 | 86.99±7.23 |
|  | E-158 | 0.01±0.02 | 0.00±0.00 | 0.01±0.01 | 0.00±0.00 | 0.00±0.00 |
| *TOS8* | KF-7 | 14.01±1.41 | 26.89±2.94 | 17.75±2.59 | 149.27±10.94 | 130.45±10.05 |
|  | E-158 | 0.00±0.00 | 0.03±0.01 | 0.00±0.00 | 0.00±0.00 | 0.00±0.00 |
| *YOL162W* | KF-7 | 26.21±2.04 | 43.25±2.61 | 70.20±6.57 | 21.15±3.14 | 9.89 ±4.34 |
|  | E-158 | 0.04±0.08 | 0.00±0.00 | 0.04±0.08 | 0.00±0.00 | 0.00±0.00 |
| *YOR012W* | KF-7 | 14.48±1.94 | 18.18±3.18 | 19.71±1.34 | 26.01±4.71 | 18.08±3.83 |
|  | E-158 | 0.00±0.00 | 0.00±0.00 | 0.00±0.00 | 0.00±0.00 | 0.00±0.00 |
